# Controlled self-organization of polymer nanopatterns over large areas

**DOI:** 10.1038/s41598-017-09463-z

**Published:** 2017-09-05

**Authors:** Ilknur Hatice Eryilmaz, John Mohanraj, Simone Dal Zilio, Alessandro Fraleoni-Morgera

**Affiliations:** 1 FlexTronix Laboratory, Dept. of Engineering and Architecture, University of Trieste - V. Valerio 10, 34100 Trieste, Italy; 2 IOM-TASC CNR, Strada Statale 14 km 163, 5–34149 Basovizza, Trieste, (TS) Italy; 3 Present Address: Advanced Functional Polymers Laboratory, Department of Macromolecular Chemistry I, University of Bayreuth, 95440 Bayreuth, Germany

## Abstract

Self-assembly methods allow to obtain ordered patterns on surfaces with exquisite precision, but often lack in effectiveness over large areas. Here we report on the realization of hierarchically ordered polymethylmethacrylate (PMMA) nanofibres and nanodots over large areas from solution via a fast, easy and low-cost method named ASB-SANS, based on a ternary solution that is cast on the substrate. Simple changes to the ternary solution composition allow to control the transition from nanofibres to nanodots, via a wide range of intermediate topologies. The ternary solution includes the material to be patterned, a liquid solvent and a solid substance able to sublimate. The analysis of the fibres/dots width and inter-pattern distance variations with respect to the ratio between the solution components suggests that the macromolecular chains mobility in the solidified sublimating substance follows Zimm-like models (mobility of macromolecules in diluted liquid solutions). A qualitative explanation of the self-assembly phenomena originating the observed nanopatterns is given. Finally, ASB-SANS-generated PMMA nanodots arrays have been used as lithographic masks for a silicon substrate and submitted to Inductively Coupled Plasma-Reactive Ion Etching (ICP-RIE). As a result, nanopillars with remarkably high aspect ratios have been achieved over areas as large as several millimeters square, highlighting an interesting potential of ASB-SANS in practical applications like photon trapping in photovoltaic cells, surface-enhanced sensors, plasmonics.

## Introduction

The controlled realization of nanopatterns on a surface is a theme of capital importance for nanotechnology. For example, this ability is of primary importance in micro/nanofluidics^1^ or in understanding the effect of a substrate topography on the development of cultured stem cells^2^, or in plasmonics-based devices^3^. While it is possible to realize surface nanopatterns with impressive precision by the so-called “top-down” approach, consisting essentially in hard lithography^4^, in recent years researchers gained some ability to create complex nanostructures using the “bottom-up” approach, thanks to self-assembly processes. One of the most successful examples in this field is the use of self-assembled monolayers (SAMs), that allows to uniformly modify the chemical nature of surfaces over large areas^5^. However, in order to organize finely resolved nanopatterns on surfaces, this approach still needs the support of lithography, which helps to direct the SAMs deposition over lithographically selected zones of the substrate. To this end, traditional lithography can be exploited^6^, but soft lithography is usually preferred, due to its simplicity, versatility and ability to deliver extra-fine features^7, 8^. Another self-assembly phenomenon leading to surface organization is the spatial nanoconfinement of the material to be patterned^9^, but also with this strategy one needs to use soft lithography techniques to obtain well defined nanopatterns, rather than solely relying on self-assembly. A general drawback of soft lithography is that it cannot deliver uniform and finely resolved patterns over large areas, due to known problems of deformation of the elastomeric stamp used for reproducing the imprinted nanometric features^10^. For achieving controlled organization at the nanoscale of large surfaces it is then possible to resort to wet processing-based methods, that can create uniform films of materials capable of self-assembly, like for example block copolymers^11^ or nanoparticles^12^, or (less often) combinations of different materials^13^. Unfortunately, none of them is able to deliver at the same time low cost, rapidity and versatility in terms of developed nanopatterns. Another possible route to large area patterning involves dewetting phenomena, that exploit the surface energy differential between a high surface energy liquid/solution and a low surface energy substrate. If the latter is properly patterned with appropriately low surface energy zones, an ordered dewetting of the high surface energy liquid/solution from the low surface energy parts of the substrate will occur. This strategy has been used for realizing macroscopic ordered patterns based, for example, on porphirine derivatives^14^ or on nanoparticles to be used as catalysts for carbon nanofibers growth^15^, and even polymers^16, 17^. However, also in this case a preliminary substrate patterning step is required prior to obtain the desired surface organization. It is possible to obtain large area surface organization based on thin polymer films dewetting (induced by temperature increase) even in absence of this preliminary surface patterning step^18^, but in these cases the obtained surface features are not ordered.

**Figure 1:**
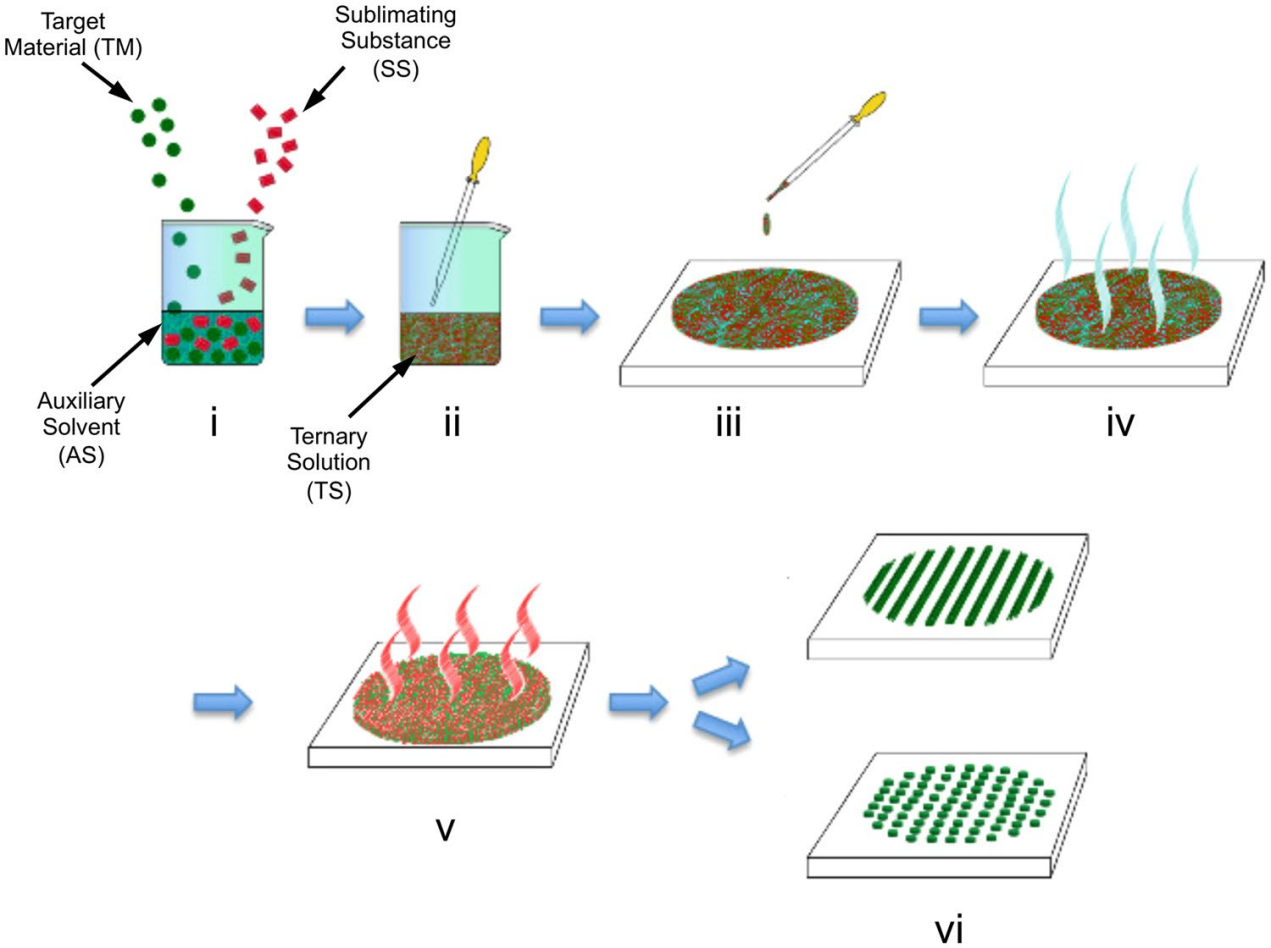
Scheme of the ASB-SANS procedure, step-by-step. (i) The TM, SS and AS are mixed together in a convenient vessel; (ii) a homogeneous, liquid TS is formed; (iii) the TS is cast onto a substrate; (iv) the AS evaporates, leaving on the substrate a solid solution formed by the crystallized SS that acts as a solid solvent for the TM; (v) the SS sublimates away; (vi) TM nanostructures of different topologies, depending on the composition of the TS, are obtained.

Recently we described a novel wet-processing method able to produce in a few minutes nanopatterns over large areas, effective on both polymers and carbon nanotubes, with no need for the use of any additional pattern-directing technique. The method is termed ASB-SANS (Auxiliary Solvent-Based-Sublimation Aided NanoStructuring)^19, 20^ and is based on the use of a Ternary Solution (TS) incorporating the material to be nanopatterned (Target Material, TM), an Auxiliary Solvent (AS) and a Sublimating Substance (SS). The TS can be deposited onto a substrate by means of simple techniques (like drop-casting), and after the complete solvents (both the SS and the AS) evaporation/sublimation, ordered nanopatterns are found on the substrate (Fig. 1). The method is effective in delivering nanofibres, straight or branched, out of different TMs, like polymers (poly(methyl methacrylate) - PMMA^19^, poly(3-hexylthiophene) - P3HT^20^ - and poly(L-lactic acid) - PLLA^21^) or carbon nanotubes^19^, and allows some control over the developed topology, in terms of fibre branching degree and developed fibre widths. The so-obtained patterns are developed over cm^2^ of substrate in minutes from the TS deposition and with inexpensive procedures, equipment and chemicals.

Here we describe an extensive investigation over the effect of changing the composition of the Ternay Solution over topology and size of ASB-SANS-generated PMMA nanopatterns. In particular, we show that carefully tuning the TS composition results in a controlled transition from continuous nanofibres to aligned nanodots, producing a range of intermediate topologies. A ternary diagram aimed at clarifying the relations between the various TS compositions and the resulting nanopatterns topologies is reported. Moreover, the fibres/dots lines lateral width and the inter-pattern distances are analysed with respect to the TS composition, and these variations are commented in the frame of currently accepted semiempirical models for macromolecular chains mobility in a liquid medium. On the basis of the observed experimental evidences, a qualitative explanation of the self-assembly phenomena originating the observed nanopatterns is given. Finally, we demonstrate that the ASB-SANS-generated PMMA nanopatterns can be used for creating arrays of high aspect ratio silicon nanopillars over areas of several thousands of μm^2^. In particular, ASB-SANS-fabricated arrays of PMMA nanodots have been used as lithographic mask for a silicon substrate, in turn submitted to Inductively Coupled Plasma-Reactive Ion Etching (ICP-RIE). The result of this process are described and commented, highlighting the potential of ASB-SANS for different applications.

## Results

### Nanopatterns realization

The ASB-SANS procedure is depicted in Fig. 1. ASB-SANS takes advantage of an organic crystal able to sublimate (a Sublimating Substance, SS), which is used as an easily removable templating matrix for the material to be structured/patterned (Target Material, TM), for which the SS is a solid solvent^19^. In order to ensure a better manipulation of the SS/TM system that will originate the nanopatterns, both materials are mixed with an appropriate Auxiliary Solvent (AS), able to solvate both the TM and the SS. In this way it is possible to realize a Ternary Solution (TS) composed by the three Auxiliary Solvent, Sublimating Substance and Target Material components, liquid at room temperature and easy to handle. Moreover, the AS is chosen among those with a low boiling point, so as to allow its evaporation before the sublimation of the SS (vide infra). In the here analysed Ternary Solution, the SS is para-dichlorobenzene (PDCB), a simple aromatic molecule (one benzene ring bearing two symmetrically arranged chlorine atoms). PDCB is fully soluble in many organic solvents, including chloroform (CHCl_3_), that was hence used as AS due to its comparatively low boiling point (61.5 °C). Poly-methyl methacrylate (PMMA) was used as Target Material, given its solubility in both CHCl_3_ and PDCB. PDCB forms beautiful needle-like crystals in normal conditions (i.e., ambient pressure and room temperature of 25 °C; monoclinic α-phase, space group P21/a; number of molecules per unit cell, Z = 2^22^). When this liquid mixture is deposited on the substrate, at first the AS and then the SS leaves the TS, as shown in Fig. 1 (steps iv and v, respectively), leaving on the substrate a well-defined pattern, whose topology is dictated by the Ternary Solution composition.

### Nanopatterns topology systematic changes

**Figure 2:**
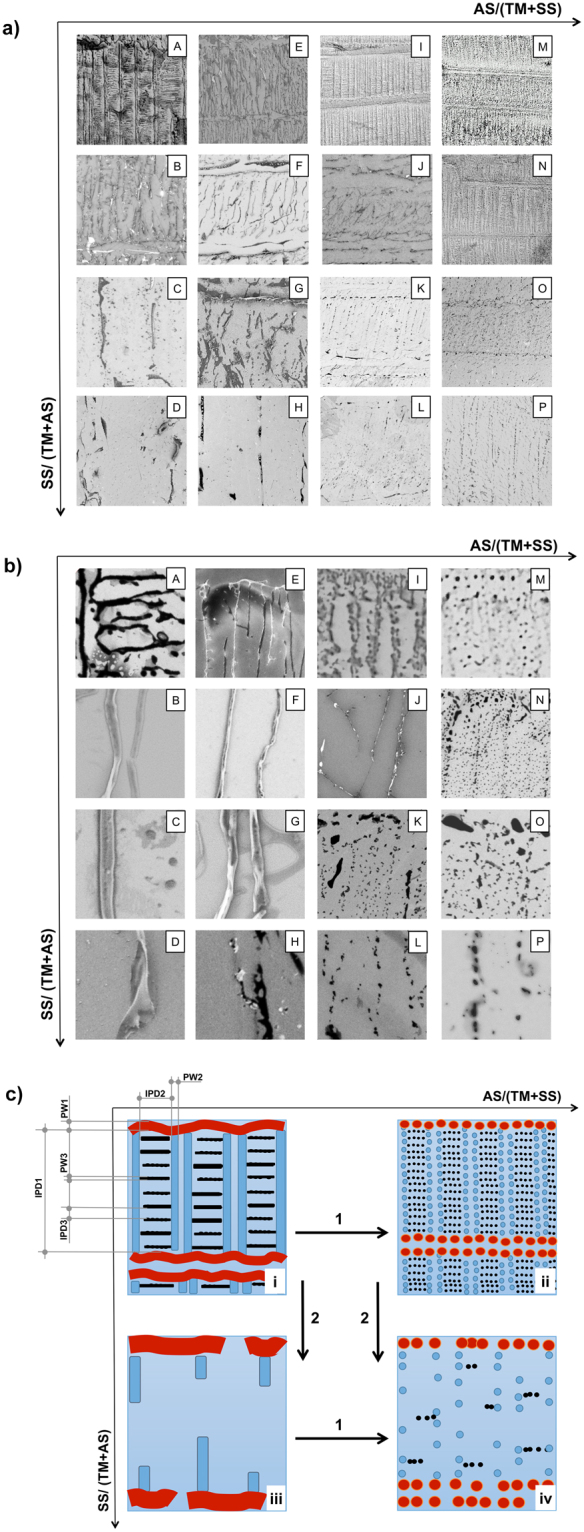
(**a**) SEM images of PMMA patterns developed onto Si/SiO_x_ substrates after the deposition of the 16 Ternary Solutions described in Table 1. Each photo is 91.8 × 91.8 μm, and is labelled according to the ID of the TS from which the pattern has been generated, as given in Table 1. (**b**) SEM images of the same PMMA patterns shown in Fig. 2a, accordingly labeled, with a higher magnification (each photo is in this case 10 × 10 μm). Only a few primary patterns have been imaged here (Panels E, I, M, N, K, O), as this would have implied a marked loss of information about the secondary and tertiary nanostructures (primary structures have sizes in the range of a few hundreds of nm-3 μm, while secondary-tertiary ones have sizes in the range on hundreds-tens of nm). Higher magnifications were not always possible to sudden damage experienced by the insulating PMMA under the electron beam at magnifications higher than 10.000x, hence for reasons of homogeneity all the images have been left to a relatively low magnification. (**c**) Cartoon illustration of the general trends in the topology development observed in Fig. 2a upon changing the TS composition. In panel (i) the labels have the following meaning: IPD stands for Inter-Pattern Distance, and the following number refers to primary (1), secondary (2) and tertiary (3) structures; PW stands for Pattern Width, with its numbering having the same meaning of the IPD notation. See text for more details.

In order to explore the effect of the Ternary Solution compositional change on the developed topology, sixteen TS types having the compositions described in Table 1 were realized according to the Methods sections, and deposited onto Si/SiO_x_ plates. The topologies deriving from each different TS are shown in Fig. 2. In Fig. 2a SEM images are arranged in a 4 × 4 matrix of equal size photos, each having about 92 × 92 μm side and labelled as the starting Ternary Solution sample IDs described in Table 1. This low magnification has been chosen to evidence the satisfactory homogeneity of the patterns and the main topological variations occuring in the patterns upon changes in the TS composition at the long range. In Fig. 2b a higher magnifications of the photos shown in Fig. 2a, labeled corresponding to Fig. 2a, are shown. In this case the SEM images have a size of 10 × 10 μm. In order to facilitate the understanding of the effect of TS composition variation, the typical Target Material topologies found at the four corners of the matrices shown in Fig. 2a,b are summarized in Fig. 2c, where panel (i) shows the typical topology observed in samples of type A, panel (ii) that of samples of type M, panel (iii) that of samples of type D and panel (iv) that of samples of type P.

**Table 1:** Composition of the prepared samples (the Sample ID identifies both the composition of the starting solutions and the topological aspect of the nanostructures imaged in Fig. 2a,b).

TS components amounts	Sample ID															
	A	B	C	D	E	F	G	H	I	J	K	L	M	N	O	P
Stock solution (μL)	75	75	75	75	50	50	50	50	25	25	25	25	10	10	10	10
AS (μL)	25	25	25	25	50	50	50	50	75	75	75	75	90	90	90	90
SS (mg)	7.5	15	30	75	5	10	20	50	2.5	5	10	25	1	2	4	10

With reference to Fig. 2c, the patterns present a hierarchical structure arranged in primary (red, large motives), secondary (cyan motives, generally thinner than the primary ones and stemming from the latter) and tertiary (deep blue motives, thinner than the secondary ones, and generally stemming from the latter) level structures. Each hierarchical level is developed orthogonally to the previous one, an arrangement already found in previous studies^19–21^. In all the panels, the different topologies are organized in the frame of the same Cartesian diagram related to the Ternary Solution composition from which the patterns have been developed. In particular, the horizontal axis labelled with the AS/(SS + TM) parameter represents growing Auxiliary Solvent contents in the TS from left to right, and the vertical axis labelled with the SS/(AS + TM) represents growing Sublimating Substance contents in the TS parameter, from top to bottom.

In Fig. 2a,b it is possible to see that the increase of the Auxiliary Solvent concentration (horizontal direction) leads to a progressive transition from continuous patterns to disrupted ones, delivering a well defined dotted morphology at the highest AS/(SS + TM) ratios. In particular, this transition is recognizable comparing the series of panels A-M, B-N, C-O, D-P, and is is better clarified in the cartoon of Fig. 2c, along the same horizontal direction. Also variations in the main geometrical parameters characterizing the patterns topology occur; in particular, the Inter-Pattern Distance (IPD in Fig. 2c, panel (i)) at same-hierarchical level motives decreases. Along the dotted patterns dots conserve a notable degree of alignment, and a relatively small dispersion of the dots’ size, at all hierarchical levels of the motives, is also observed.

The increase of the Sublimating Substance concentration causes several topological changes in the ASB-SANS-developed patterns, visible along the vertical axis of the diagram of Fig. 2a,b: (i) the higher orders of patterns are almost suppressed (tertiary level) or greatly diminished in occurrences (secondary level), for both fibrillar and dotted patterns, as can be observed comparing vertically arranged panels A–D, E–H, I–L, M–P; (ii) the size of the patterns (Pattern Width, PW in Fig. 2c, panel (i) evolve from smaller (A, E, I, M) to larger (D, H, L, P) structures. Again, this is verified for both fibrillar and dotted patterns; (iii) Inter-Pattern Distances tend to become greater, once more irrespectively of fibrillar or dotted patterns (compare columns A–D, E–H, I–L, M–P). These trends are exemplified more clearly in Fig. 2c.

**Figure 3:**
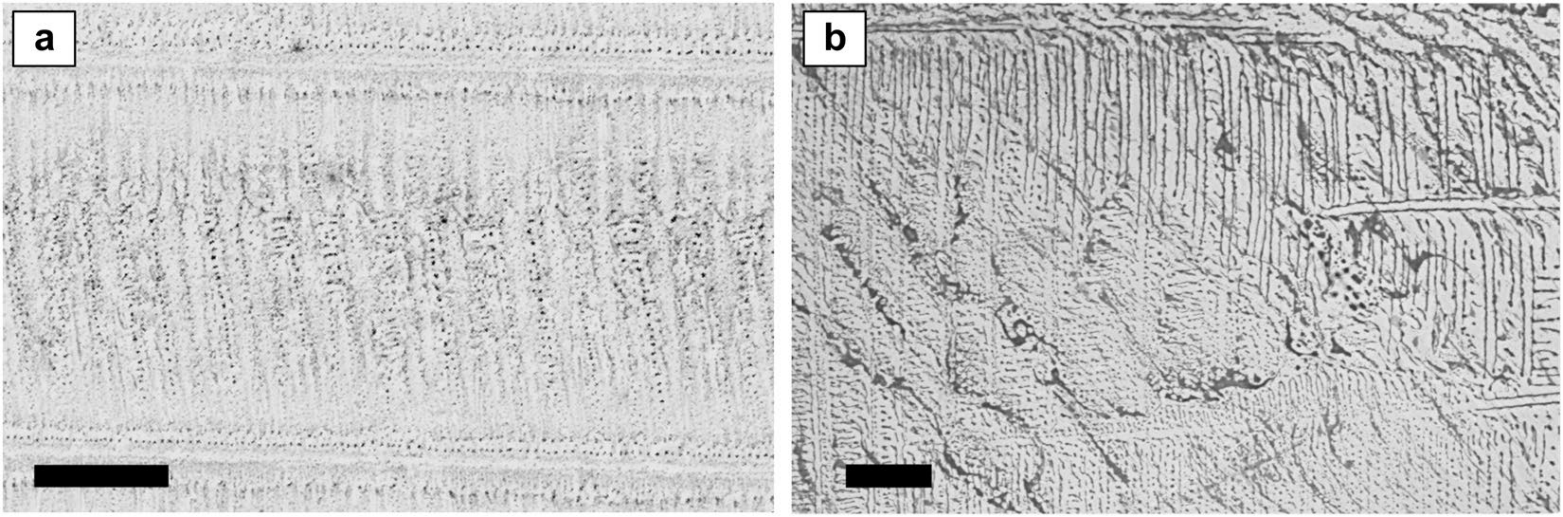
(**a**) low magnification SEM image of a PMMA pattern obtained using the TS of type M (see Table 1), evidencing the good uniformity of the developed nanopatterns over considerably large areas (approx. 10.000 μm^2^; scale bar = 20 μm); (**b**) SEM image showing a gradual transition from a continuous fibre pattern to a dotted one. Scale bar = 10 μm.

Interestingly, the dotted morphology (column M-P in Fig. 2a,b) can be developed over extremely long lines, up to several tens of microns, still conserving little dispersed dots diameters, well below one micron, as is visible in Fig. 3a, which shows a sample obtained from a Ternary Solution of type M. This is verified at each level of the patterns hierarchy. This type of PMMA morphology can be useful in fields like lithography, for example for realizing large arrays of nanostructured silicon surfaces. In some samples the developed patterns presented a mixed morphology, including continuous and disrupted fibres, as well as aligned dots (Fig. 3b). This pattern topology variability within the same sample is attributed to local variations of Target Material concentration or inhomogeneous SS/TM layer thicknesses, due to non-equilibrium mass transport phenomena occurring inside the drop cast Ternary Solution. In turn, this latter occurrence is most likely due to the fact that the ASB-SANS procedure has been conducted in non strictly controlled conditions. In fact, no particular equipment for controlling the environmental humidity has been used, and the TSs were manually cast onto the substrates, a procedure extremely sensitive to even minute variations, as for example the angle with which the pipette approaches the surface, the distance between the surface and the pipette, or possible sudden micro air flows in the surroundings of the sample. These factors influence the development of the PDCB crystals, which in turn appear to be the templates that dictate the final size, orientation and morphological regularity of the nanopatterns (vide infra).

As several nanopatterns were imaged by SEM more than one time (the first time just after their obtainment, the second or third time after periods ranging from three to twelve months), the produced patterns appear to be thermodinamically stable.

### Visualization of the topology variation in the TS composition space and assessment of dimensional patterns parameters

**Figure 4:**
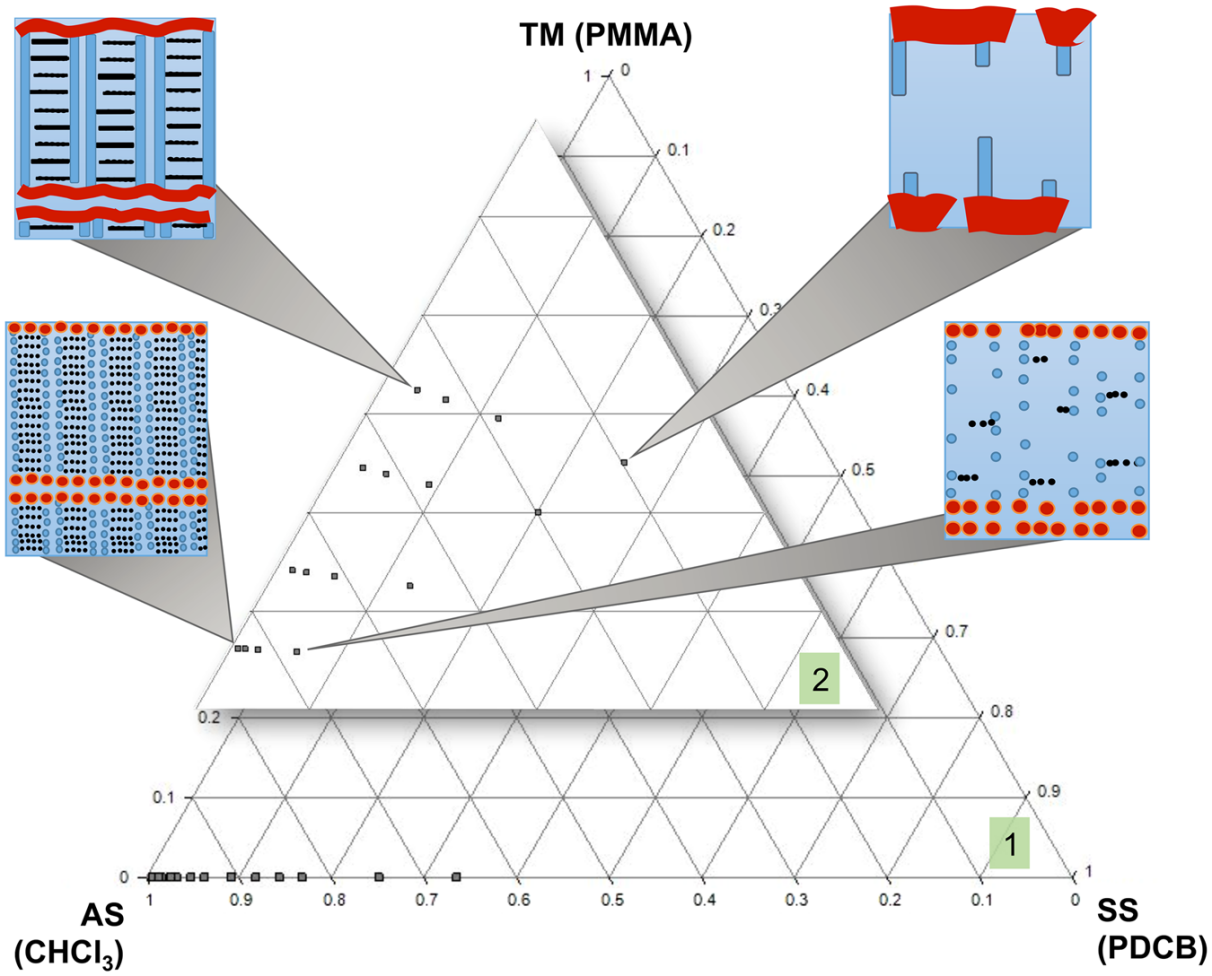
Ternary diagram showing the compositions of the analyzed Ternary Solutions, and their relation with the observed patterns morphologies (in the inset 2 the TS compositions is not exact and the diagram is slightly distorted to allow to appreciate the effect of the TS compositional changes over the developed patterns topology).

The influence of the relative concentrations of the Ternary Solution components on the developed patterns topology can be visualized on a ternary diagram, as shown in Fig. 4. In this diagram the positions of the data points reported in the inset (**2**) have been slightly changed with respect to the actual compositional values - refer to Table 1 - to better appreciate the effect of changes in the TS composition on the patterns topology and to avoid the “squeezing” on the diagram base of all the analyzed TS compositions, visible in the main diagram (1)). The most continuous patterns are found at relatively high concentrations of Target Material (upper left part of the diagram); going towards progressive dilution of the TM in the TS results in disruption of the patterns, originating either discontinuous fibres (upper-right part of the diagram) or aligned dots (lower part of the diagram). The increase of Auxiliary Solvent results in the formation of aligned dots arrays instead of continuous fibres (lower part of the diagram).

The Pattern Width, PW, and the Inter-Pattern Distance, IPD, have been measured as depicted in Fig. 2c, panel i), using the collected SEM images with the aid of image processing software, for 20 to 50 different samples per each relevant pattern type. This has been done at all pattern levels (i.e., primary, secondary and tertiary) and irrespective of whether the patterns were continuous or dotted. Where the pattern level was not clearly recognizable or was absent, as in the most TM-diluted Ternary Solutions, these measurements have not been made﻿.

In terms of numerical values, the range of Pattern Widths (intended as lateral values, and not as vertical ones, i.e. not as patterns’ thickness) and Inter-Pattern Distances found in the whole set of examined samples (i.e., going from A to M) is relatively well defined, and always confined within about one order of magnitude for each hierarchical level (i.e., at Primary, Secondary and Tertiary level), even though some overlap between different hierarchical levels is observable. This finding is summarized in Table 2. In more detail, changing the composition of the Ternary Solution from which the samples A-M are originated results, for Pattern Widths, in values between about 0.3 (minimum average value upon the analyzed samples) and 3 (maximum average value upon the analyzed samples) μm for the Primary level patterns. For the Secondary patterns, this range was 0.2–1.4 μm; for the Tertiary (when present) in the range 0.07–0.6 μm. With respect to the Inter-Pattern Distance, for Primary patterns the range is 27–571 μm; for Secondary ones, 3–60 μm; for Tertiary ones, 0.5–8 μm.

**Table 2:** Minimum and maximum values of Pattern Widths and Inter-Pattern Distances found in the examined samples A-M (i.e., upon changing the starting Ternary Solution composition) for primary, secondary and tertiary patterns.

	Pattern Width (PW, μm)		Min-Max Inter-Pattern Distance (IPD, μm)	
	Min	Max	Min	Max
Primary Patterns	0.36 ± 0.08	2.9 ± 0.7	28 ± 8	570 ± 80
Secondary Patterns	0.21 ± 0.08	1.4 ± 0.6	3.2 ± 0.7	59 ± 15
Tertiary Patterns*	0.08 ± 0.01	0.6 ± 0.1	0.5 ± 0.1	7.6 ± 0.5

*For tertiary patterns some tiny features were not measured, as they were often ill-defined and/or damaged under the electron beam of the SEM.

## Discussion

### Relations between the nanopatterns topology and the composition of the starting Ternary Solution

**Figure 5:**
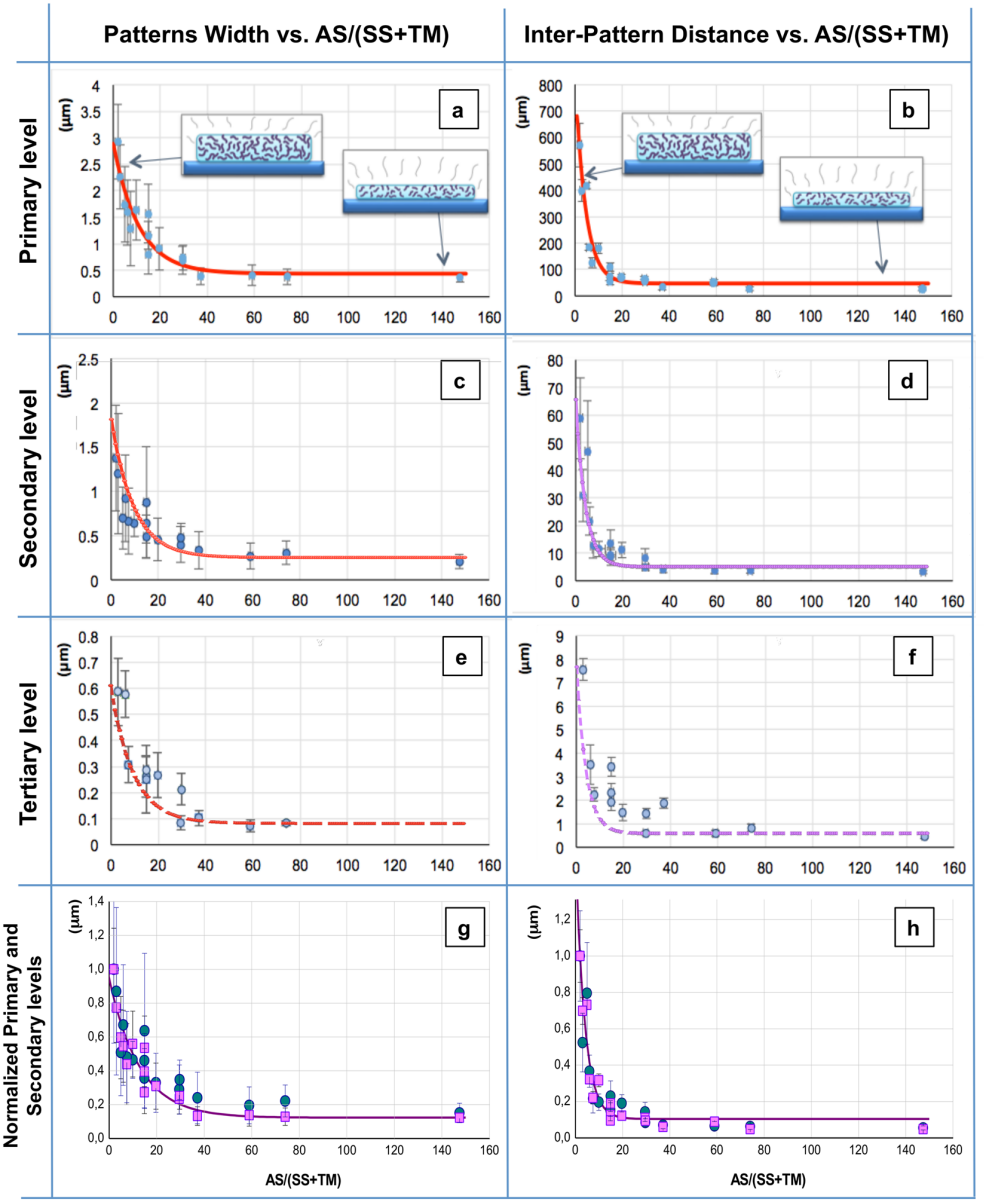
(**a**–**f**) Plots of the Pattern Widths (left column) and Inter-Pattern Distances (right column) arranged along three rows (Primary level (**a**,**b**), row 1; Secondary level (**c**,**d**), row 2; Tertiary level (**e**,**f**), row 3) in function of the AS/(TM + SS) ratios for the patterns obtained from the 16 different realized TSs. Each data point is the result of 20 + different measurements, and the error bars show the data standard deviation. The red interpolating curves in the two plots of row 1 are the best fits for the given data points of the Primary levels; the purple continuous interpolating curves at the Secondary levels are exactly the same fits of the Primary levels, just graphically overlapped onto the collected data points (hence they are not actual fits). The purple dashed interpolating curves at the Tertiary level are again the same graphical respective fits of row 1, and are overlapped to actual data as a guide to the eye. In the insets of the Primary levels panels (row 1): qualitative sketches of the solid SS/TM layer thickness obtained at different AS/(TM + SS). (**g**,**h**) Normalized Primary (pink squares) and Secondary (blue circles) levels Pattern Widths (**g**) and Inter-Pattern Distances (**h**). The fitting curves (purple plots) are calculated for the Primary levels of the patterns (see text for details).

The above reported topological data, in terms of both the type of developed pattern (i.e., fibers or dots) and their lateral size and relative lateral distance, as measured by SEM images analysis, point to some form of ordered PMMA assembly occurring during the ASB-SANS process. In order to get some insight into this phenomenon, we plotted the quantitative topological data (Pattern Widths and Inter-Pattern Distances) vs the AS/(TM + SS) parameter. These plots (Fig. 5, where the standard deviation for each datum is shown as error bars) bring to clear evidence that both PWs (being them continuous or disrupted fibres, or dots) and IPDs decrease with the increase in AS/(TM + SS), at each considered hierarchical level (Fig. 5a,c,e for PWs and Fig. 5b,d,f for IPDs). Both these quantities reach a lower plateau at the highest AS/(SS + TM) ratios tested.

At the Primary level, for both the PW and the IPD, it is possible to satisfactorily fit the data points with first order exponential decay curves of the type *y* = A e^−(1/B)*x*^ + *y*_*0*_ (Fig. 5a,b). For the Pattern Widths, the fit equation is *y* = 2.465 e^−(1/10.84)*x*^ + 0.4360, with an adjusted R^2^ = 0.889, while for the Inter-Pattern Distance the fit equation is *y* = 793.2 e^−(1/4.377)*x*^ + 47.13, with an adjusted R^2^ = 0.913. For both the Pattern Widths and the Inter-Pattern Distances, a simple graphical translation of the respective Primary level fitting curves over the corresponding Secondary level plots appears to properly fit the data points from a qualitative point of view, (Fig. 5c,d, continuous curves), while the same procedure applied to the respective Tertiary levels data delivers a less satisfactory visual fit (Fig. 5e,f, dashed curves). In order to check whether this apparent coherence of the fitting curves over data coming from different hierarchic levels is an actual feature of the investigated systems or not, the data related to the Primary and Secondary levels (both PWs and IPDs) have been normalized. The Tertiary level patterns parameters have not been normalized because at these really low sizes the patterns are often ill-defined, hence some data points were missing and a normalization procedure coherent with the ones conducted for the Primary and Secondary levels was not possible. The normalized data points related to the Primary levels have been hence fit with first order exponential decay curves (Fig. 5g,h, purple curves), delivering very good adj. R^2^, higher than 0.9 (see Table 3) for both the Pattern Widths and the Inter-Pattern Distances. When the same fitting curves parameters A and B have been applied to fit the corresponding Secondary levels (except for the offset values y_0_, left floating to allow fit convergence) the respective adj. R^2^ resulted to be excellent for the Inter-Pattern Distances (adj. R^2^ = 0.93), and less good but still satisfactory (adj. R^2^ = 0.811, Table 3) for the Pattern Widths. A relatively high standard deviation of the latter data was found, due to the poorly defined features at the smallest TM concentrations (see Fig. 2b).

**Table 3:** Fitting curves parameter for normalized pattern characteristics at the primary and secondary hierarchic levels.

Parameter	Normalized Pattern Widths		Normalized Inter-Pattern Distance	
	Primary level	Secondary level	Primary level	Secondary level
A	0.8223	0.8223	1.479	1.479
B	12.76	12.76	3.821	3.821
y_0_	0.1230	0.1852	0.1040	0.0839
Adj. R^2^	0.915	0.8111	0.932	0.931

The fact that the topological features (Patterns Widths and Inter-Pattern Distances) of both the Primary and Secondary hierarchic levels can be fit with essentially the same exponential decay curves strongly suggests that the phenomenon at work in the formation of these features can be the same at all the considered patterns dimensional scales (with a certain degree of speculation for what about the tertiary levels). It is worth to recall here that from the mid-‘50 s a seminal work of Zimm^23^ led to the development of a number of models describing the diffusion of polymeric chains within diluted liquid solutions. Several of these models are based on considering the macromolecule as a collection of connected segments traveling in the diluted solution. In this frame, the description of the motion of the macromolecule includes an exponential decay dependence of the distance travelled by two ends of the chain with respect to the time elapsed between one discrete step movement of a chain segment and the subsequent one^24, 25^. In our case, a number of observations suggests a parallel between general Zimm-like models and the trend found in Pattern Width and Inter-Pattern Distances, as follows: i) both the Patterns Widths and the Inter-Pattern Distances can be viewed as proxies of the PMMA (our Target Material, TM) chains mobility within the matrix constituted by the Sublimating Substance SS (because the more the chains are mobile, the more they will be able to migrate within the SS matrix to form larger patterns or more distant patterns); ii) in each considered Ternary Solution the TM diffusion proceeds within a diluted solid solution (between 0.1 and 1% in TM/SS, weight percentage, see Table 1, where the solvent is the Sublimating Substance left on the substrate after the Auxiliary Solvent - AS - evaporation); iii) the AS/(SS + TM) parameter can be seen as a measure of the time available to the PMMA to travel through the Sublimating Substance matrix once the Auxiliary Solvent has left the system. In fact, the more AS is present in the Ternary Solution, the less SS there is, meaning that the SS/TM layer left on the substrate after the AS evaporation will be thinner. This occurrence will leave less time to the Target Material for moving within the solid matrix (see Fig. 5 and vide infra for more detail); iv) the Pattern Width and the Inter-Pattern Distance in function of the AS/(SS + TM) ratio, which is substantially a proxy of the time available to the Target Material to diffuse within the Sublimating Substance matrix before this latter is sublimated away, are linked by exponential decay relations at the Primary and Secondary level (it is not possible to assess the same occurrence at the Tertiary level with the available data). On these grounds, we are tempted to draw a parallel between Zimm-like models for macromolecular motion within liquid, diluted solutions and our case of macromolecular motion within solid (PDCB, our Sublimating Substance), diluted solutions. In any case, the above described exponential decay correlation, obtained despite the relatively large distribution of the examined dimensional parameters of the nanopatterns, evidenced in the Discussion section (see Table 2 and related text), suggests that the ASB-SANS procedure is overall robust and has some potential for delivering rather homogenous patterns over thousands of μm^2^, as it is possible to appreciate from Figs 2a, 3a and S1 (available as Supporting Information). In particular, more controlled Ternary Solution deposition methods, like operation in a glove box with an automated solution dispenser, could deliver a much higher degree of control over the final pattern regularity and definition.

### Rationale for the PMMA (Target Material) mobility within the solid, crystalline PDCB (Sublimating Substance) matrix

There are several possible mechanisms that can grant the PMMA molecules the mobility within the solid crystalline PDCB matrix necessary to form the observed nanostructures. A first possibility is that the PMMA solute induces a PDCB melting temperature decrease, creating a liquid solution that would allow PMMA to easily diffuse within the PMMA-PDCB mixture. However, previous studies conducted on the same PDCB (SS)/CHCl_3_ (AS)/PMMA (TM) system evidenced that upon the Auxiliary Solvent evaporation the TM/SS solid solution form clearly defined and regular crystals (see Fig. 3 of ref. 19), at TM/SS concentrations higher than those reported in this work (i.e., 5–0.7% for ref. 19 vs 1–0.1% of this work, percentages in w/w). Therefore, the here reported TM concentrations do not cause macroscopically detectable liquefaction of the PDCB solid structure, nor PDCB crystal structure disruptions. Indeed, we observed this latter occurrence with TM/SS ratios higher than 7% w/w (data not reported), but also in this case the PDCB/PMMA solid solution remained solid, though losing its crystallinity. In the here discussed case, the overall amount of PMMA present in the PDCB matrix, ranging from 0.1 to 1% w/w, would be hardly sufficient to cause a macroscopically detectable melting temperature decrease. In addition, all the reported experiments have been carried out thermostating the substrates onto which the Ternary Solutions have been deposed at 23 °C, never reaching temperatures approaching the melting temperature of PDCB, which is around 53 °C. Another similar mechanism that could affect the crystallinity of the solid PDCB matrix, creating the conditions for the observed PMMA mobility, is the possible presence of residual Auxiliary Solvent in the PDCB crystals. We tend to exclude this possibility, at least at the macroscopic level, for the same reasons previously mentioned, i.e. because impurities present in the SS/TM mixture hinder the formation of crystals, that are instead observed in each analyzed sample. A further possibility for explaining the PMMA mobility within the PDCB matrix is that different phases could be formed within the solid, crystalline PDCB (SS)/PMMA (TM) solution. In particular, it is in principle possible that in the solid solution pure PDCB phases domains are present together with PMMA phases incorporating PDCB molecules plasticizing the polymer. However, if this would be the case, at the end of the sublimation process some areas of the substrate with no trace of PMMA should have been visible, something that was never verified (see Fig. 3 and Supporting Information, Fig. S1). Moreover, no traces of residual PDCB, that could act as plasticizer in the hypothetical polymer phase, have been found in the patterns by FT-IR analyses of the samples, even in previous studies^19, 21^.

Upon these considerations, we explain the PMMA migration ability in the solid PDCB phase as due to local (at the nanoscale) disruptions of the crystalline structure of the latter, induced by the presence of the PMMA chains, foreign to the PDCB crystal structure. Possibly, also trace residues of CHCl_3_ or PDCB, present as defects in the crystalline building, could contribute to this phenomenon. This local disruption of the crystalline building caused by impurities to organic single crystals (like the PMMA within the PDCB crystalline matrix) is known since long time^26^, even though it has never been investigated at the nanoscale, and it has the potential to allow to the PMMA chains free volume enough, hence the mobility, necessary to move within the otherwise solid crystalline SS matrix, forming the observed nanostructures. Even though our hypotheses fit well all the observed findings and is supported by existing literature, specific experimental and theoretical investigations are ongoing in order to ascertain this interesting point beyond any doubt.

### Proposed mechanism underlying the patterns formation

A previous study found that the TM patterns are formed exactly where the SS crystallites were before leaving the substrate by sublimation (see Fig. 3 of ref. 19 and the video uploaded as Supporting Information for said reference). The precise alignment of the patterns with the SS crystallites supports the view that in this phenomenon the Sublimating Substance crystal acts as a template for the Target Material. In particular, it is believed that the driving force for the nanopatterns formation is provided by the progressive sublimation of the Sublimating Substance (SS), that results in the progressive change of the macromolecular external environment from a good solvent (the SS) to a bad solvent (the laboratory air that substitutes the sublimated SS), forcing the PMMA macromolecules to self-organize into the observed structures. In fact, it is known that polymeric chains exposed to non-solvents tend to self-assemble into spherical particles (coil-to-globule transition)^27, 28^, and this has been verified also for PMMA^29^. Moreover, it has been proposed that solid substrates can enhance this coil-to-globule transition via surface effects^30^. Since in our case the sublimation of PDCB results for PMMA in the passage from a good solvent (PDCB) to a non-solvent (ambient atmosphere) in presence of a solid substrate, it appears reasonable that a coil-to-globule transition is triggered by the PDCB sublimation, that actually corresponds to the passage from a good (SS) to a bad (air) solvent.

**Figure 6:**
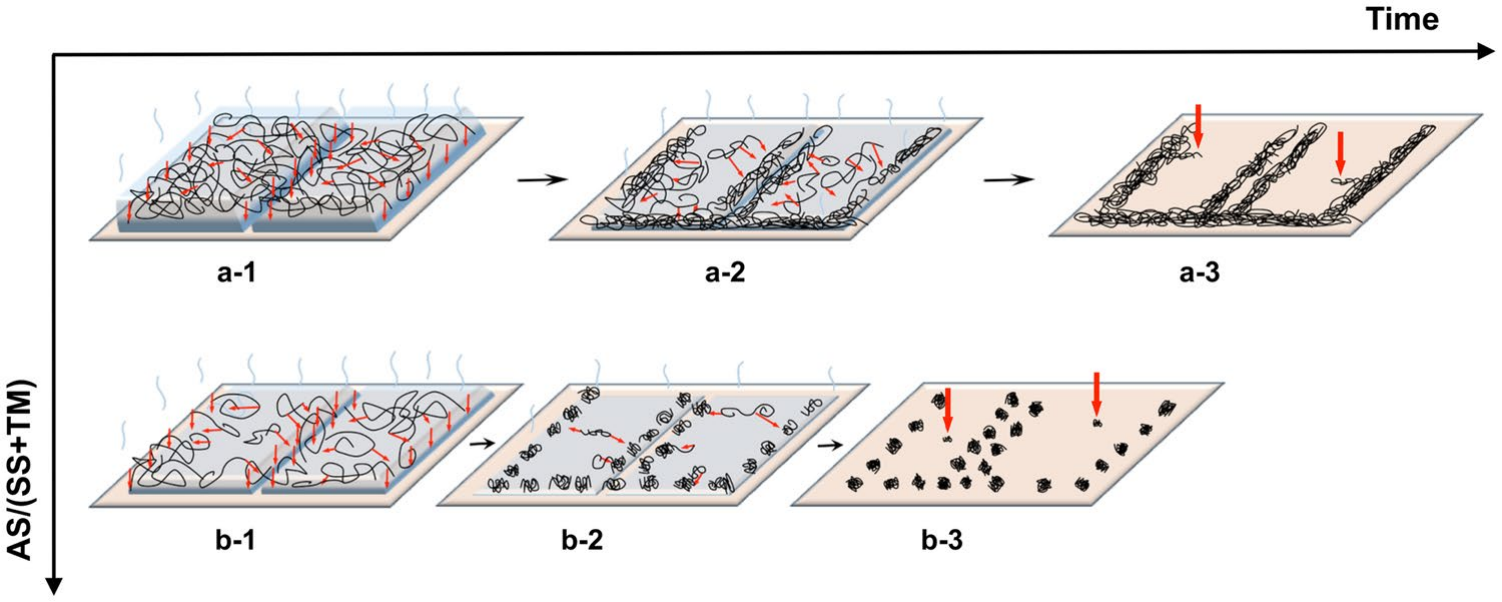
Proposed model for the observed nanostructures formation. **Row a**: nanopatterns development process ongoing using a low AS/(SS + TM) ratio. Thick SS crystals incorporating a relatively high amount of TM are formed (**a-1**); as the SS sublimates away, the TM macromolecules precipitate onto the substrate, starting from the crystallites edges (**a-2**); at the end of the process, nanofibres are formed where the initial crystallites edges were before sublimating away (**a-3**). **Row b**: nanopatterns development process ongoing using a high AS/(SS + TM) ratio. Thin SS crystals incorporating a small amount of TM are formed (**b-1**); as in these conditions the SS disappears via sublimation very quickly, the TM macromolecules do not have time enough to diffuse to the crystallites borders to self-arrange into long fibres, and start to precipitate onto the substrate as globules (**b-2**); at the end of the process, nanodots are formed along the initial crystallites edges (**b-3**). The positioning of the two rows with respect to the time axis reflects the time allowed for the TM to diffuse within the SS upon the different thicknesses of the solid SS:TM layers. See text for more details.

The topological differences found in the above described patterns upon changes in the Ternary Solution composition can be explained in terms of different SS crystals thickness obtained upon casting the different TS onto the substrate. In fact, it is clear that the transition from continuous patterns to discontinuous ones (Fig. 2a,b) occurs upon dilution of the Target Material in the ternary solution, independently from the fact that the dilution occurs upon increase of the Sublimating Substance or of the Auxiliary Solvent. However, the type of topology found on the substrate after the ASB-SANS process completion depends upon the specific composition of the Ternary Solution. In particular, when the SS/AS ratio is high, the resulting patterns are disrupted fibres (Fig. 2a,b,C,D,G,H). This is rationalized considering that when the concentration of the Sublimating Substance in the Ternary Solution is high, the solid layer resulting from the solution deposition on the substrate after the Auxiliary Solvent evaporation is thick (see Fig. 5, insets at the top left of the upper graphs, and Fig. 6, row (a). In this situation the PMMA (TM) macromolecules have a relatively long time available for diffusing through the SS solid matrix before the latter disappears by sublimation, allowing a well ordered self-assembly process that in turn leads to the formation of continuous patterns. On the contrary, when the Sublimating Substance amount in the Ternary Solution is low, it is found that the resulting patterns are aligned dots (Fig. 2a,b-M,N,O,P). In this situation the solid SS/TM layer resulting after the AS evaporation is thin (Fig. 5 - insets at the top right of the upper graphs, and Fig. 6 - row (b) and also the TM concentration is low, due to the Ternary Solution composition range here examined. The ternary diagram in Fig. 4 gives a helpful glance of the TS composition in this situation. The TM macromolecules have hence much less time available for diffusing through the solid matrix, precipitating much before being able to complete a meaningful self-assembly process, thus forming dots instead of continuous patterns. With respect to the templating effect of the Sublimating Substance crystals, it is observed that the fibres length direction always follows that of the PDCB crystallites (for the secondary patterns; for the primary patterns the relevant aligning surfaces are the domains borders, see Fig. 6 and the video uploaded as Supporting Information of ref. 19). This is attributed to the fact that the long edges of the crystallites, exposing to the atmosphere more surface than the crystallites cores, are those experiencing the faster sublimation rate. Since air is a non-solvent for the PMMA, at these edges the interface SS/air causes the polymer precipitation onto the substrate, leading to the formation of a series of PMMA nuclei aligned with the SS crystallites edges. At the end of this process the observed patterns are generated, with a phenomenon somehow resembling the formation of coffee rings^31^. The different Inter-Pattern Distances are explained again on the basis of the different thicknesses of the solid SS/TM layer. When this is thick (at low (AS/(TM + SS) ratios), the crystallite borders shrink relatively slowly, giving the Target Material time enough to diffuse to the crystallite edges. As the thickness progressively diminishes (increasing (AS/(TM + SS) ratios), a faster shrinking of the Sublimating Substance crystallites occurs, not allowing to the Target Material macromolecules time enough for diffusing through the solid matrix, hence for achieving a meaningful degree of self-assembly. The thinner the solid SS/TM layer, the more ill-defined and/or disrupted are the fibres, reaching the limit of the simplest self-assembly the macromolecules can realize, i.e. dots, when the AS concentration is very high (which means that the SS/TM solid layer left on the substrate is extremely thin). As the SS crystallite/substrate contact line-induced pinning is expected to be effective also in this situation, the resulting dots become aligned at the edges of the sublimating crystallites, delivering the same hierarchy (Primary/Secondary/Tertiary patterns) and crystallite-dictated templating effect found for the fibres. Notably, all the aligned dots belonging to the same hierarchical pattern order have relatively uniform sizes, supporting the view that the observed dotted patterns result from macromolecular diffusion-controlled self-assembly processes. For the same reasons the Inter-Pattern Distances become shorter with thinner SS/TM layers. This picture is supported also by the systematic appearance of closely aligned double fibres at the Primary and Secondary level when the SS/TM ratio is relatively low (see Fig. 2a,b, panels A,B,E,F,I,J,M,N), which points to the templating effect of the crystallites’ edges, rather than that of the crystallites’ bulk, since in that case just a single fibre should be observed. The same considerations apply to the crystallite domains borders. We explain the Tertiary patterns as originated by Target Material macromolecules that, while diffusing towards secondary patterns, have been able to form small self-assembled structures but have been “caught” in the middle of the travel by the final sublimation of the SS matrix, which caused their sudden precipitation on site (Fig. 6, cartoon a-3, red arrows). The orthogonality of the Tertiary patterns to the Secondary ones, which is compatible with an oriented motion of the Target Material chains towards the latter throughout a medium (minimization of the flow resistance^32^), is in line with this interpretation, as well as the almost complete absence of Tertiary patterns at high SS concentrations in the Ternary Solution (which means that all the TM macromolecules had enough time to diffuse and self-assemble within Secondary patterns).

The isolated and unaligned patterns (most of the times dots) often observed in between the secondary patterns are attributed to “lost” chains that did not have enough time to reach the main fibres or dots or to diffuse to a crystallite edge, and that precipitated in the middle of a crystallite, and from there onto the underlying substrate (Fig. 6, cartoon b-3, red arrows).

### ASB-SANS-generated nanopatterns as masks for direct lithography

**Figure 7:**
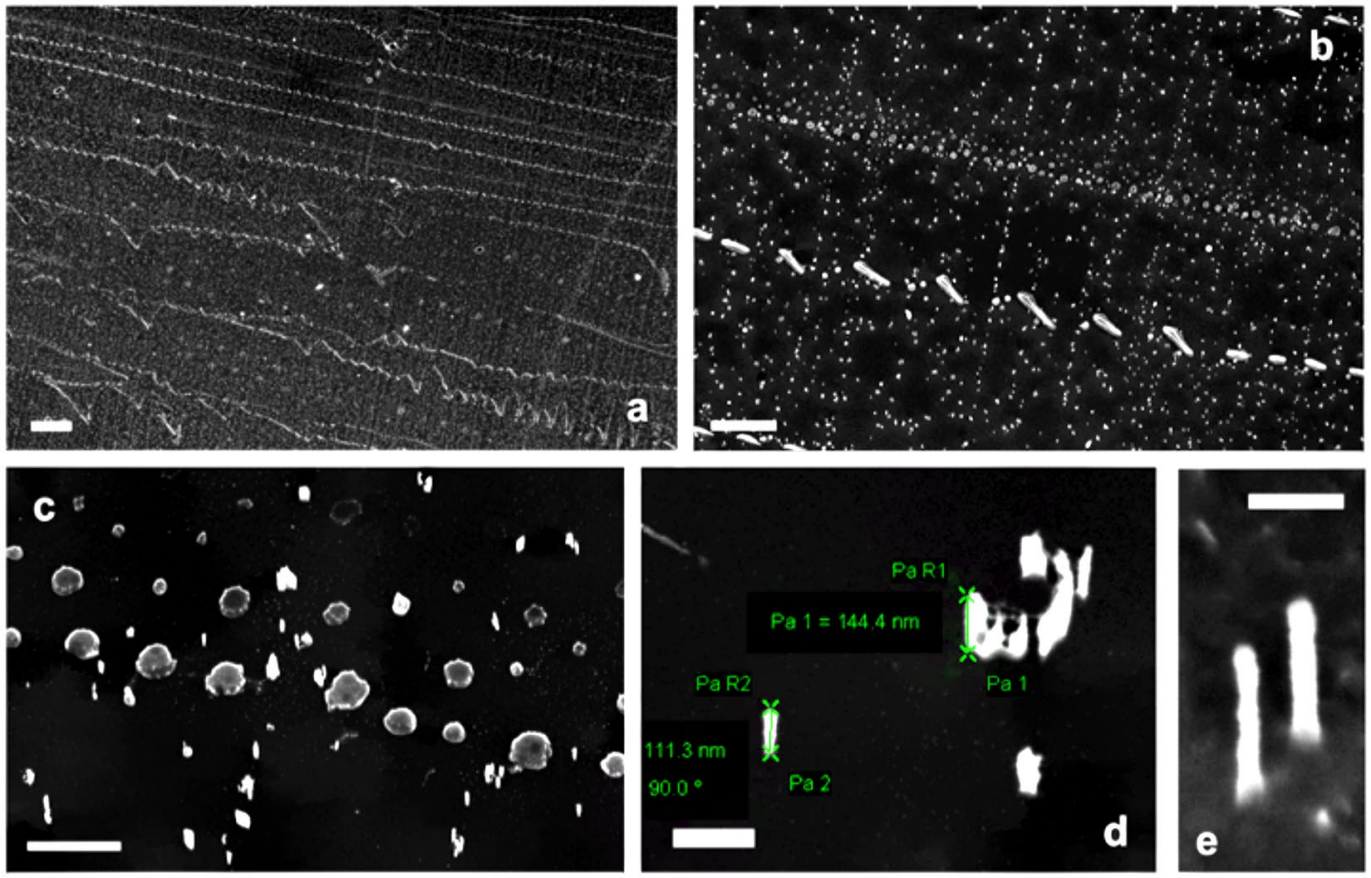
SEM images of ICP-RIE etched silicon wafers obtained by using the ASB-SANS-generated pattern of type M (Table 1) as a lithographic mask. Scale bars, respectively (**a**–**e**) 20 µm, 4 µm, 1 µm, 200 nm, 100 nm.

In order to explore potential applications of the above described ASB-SANS processes and of the ability to control the topology of PMMA nanostructures on Si/SiO_x_ substrates, we decided to use some of the developed PMMA patterns as masks for a subsequent Inductively Coupled Plasma-Reactive Ion Etching (ICP-RIE) process; in particular, we used dotted patterns developed using the Ternary Solution composition of type M. As is visible in Fig. 7, the ICP-RIE procedure carried out on these samples delivered well defined patterns in the underlying silicon, all with rather uniform heights ranging from 100 to 200 nm and with a precise vertical etch. In particular, it is possible to notice that the Primary dotted linear patterns, developed along several hundreds of thousands of μm^2^, are reproduced either with the original round shape found in the starting dotted pattern (Fig. 7a–c) or recovered as aligned nano-dashes (Fig. 7b,c). This latter occurrence can be due to the relatively high temperatures reached during the ICP-RIE process, that may go over 150 °C^33^. In fact, PMMA is known to present a lower glass transition temperature (T_g_) when patterned into nanostructures^34^, and the used PMMA has, as a bulk, a T_g_ of about 100 °C, with a melting temperature ranging between 130 and 140 °C. Moreover, from about 120 °C upwards PMMA starts to depolymerize, generating a much softer material. Therefore, we attribute this topology changes to a limited but effective local softening/melting of PMMA nanodots upon the heating imparted by the ICP-RIE treatment, that causes neighbouring dots to coalesce. With respect to the Primary nano-dots, it is observed that their diameter in the etched silicon is smaller (about 150–350 nm, see Fig. 7c) than that in the starting ASB-SANS-generated mask (about 300–600 nm, same topology of sample M) ratios, like those imaged in Fig. 2a,b-C,D,H). This is likely due to the etching process itself, which partially removes the PMMA. The Secondary dotted patterns present basically two topologies in the etched substrate: small pillars with diameters in the range of about 20 to 50 nm (again much lower than the corresponding ones in the starting PMMA patterns) and a kind of “nano-Stonehenge” systems (see Fig. 7c,d). While the former are attributed to standard, uniform dots belonging to Secondary patterns, the latter are believed to be originated by dots having a coffee ring-shape, sometimes found in samples with high AS/(TM + SS) ratios (Fig. 2b). These crater-like dots have very likely differential robustness to the ICP-RIE treatment, thus originating the observed “nano-Stonehenge” features. With respect to Tertiary patterns, it is very difficult to clearly identify them in the etched substrate. Nonetheless, due to the fact that the Secondary patterns (the “nano-Stonehenges”) are well aligned orthogonally to the Primary ones (see Fig. 7a–c), we suppose that the smallest nanopillars found (about 20–30 nm in diameter, and about 100–150 nm high), which are not clearly associable to the geometric directions of the templating PDCB, are actually due to remnants of Tertiary PMMA patterns. The tiniest columnar features have diameters down to 15 nm (Fig. 7d,e), with a very good homogeneity along all their vertical development, virtually no underetching and an aspect ratio exceeding 12:1. Such a high aspect ratio in silicon nanostructures is uncommon for ICP-RIE processes carried out using pristine (i.e., not mixed with other compounds) PMMA as a mask, especially considering the low molecular weight of the used polymer (15 KDa). Even more uncommon is the low etch rate obtained with this ASB-SANS-treated PMMA (PMMA15), calculated to be about 0.7 nm/s. In fact, when ICP-RIE is performed on masks made by PMMA of 950 KDa (PMMA950), typical etch rates are around 2 nm/s^35^. The difference between these two etch rates cannot be explained in terms of different molecular weights, since it is known that the higher the molecular weight of PMMA, the lower its etch rate^36^, while in our case we observe the exact contrary occurrence (i.e., that the ASB-SANS-treated PMMA15 is much more resistant to the RIE process than the PMMA950, despite the latter having a molecular weight two orders of magnitude higher than the former).

This notable resistance to the etching process of PMMA15 is hence attributed to a self-organization of the polymer chains imparted by the ASB-SANS process, a phenomenon already documented for P3HT, a well known semiconducting polymer^20^.

Interestingly, these results have been obtained with no particular optimization of neither the ASB-SANS procedure nor of the etching process, hence it is reasonable to think that the homogeneity of the etched layer can be greatly improved. The obtained high aspect ratio nanostructures are monolithic with respect to the underlying silicon substrate, with the consequent optimal electronic and optical continuity between the substrate and the nanostructure. From the applicative point of view, the realized patterned silicon can find useful applications, ranging from photovoltaics (as light trapping surfaces)^37^ to sensing^38, 39^. If the silicon is properly doped, also applications in plasmonics are possible^40^. Moreover, as ASB-SANS is applicable also to surfaces different from silicon (for example on glass^21^), the same nano-mask fabrication approach is conceivable over other photovoltaics-relevant materials, like TCOs^41^ or thin-films-related metals^42^. These possibilities will be explored in further, ongoing work.

In conclusion, we have shown that it is possible to control the transition from nanofibres to nanodots of PMMA in nanopatterns generated by ASB-SANS by simple variation of the Ternary Solution composition. In particular, working in the proper conditions allows to create aligned nanodots patterns over rather large areas (as wide as several thousands of μm^2^) within minutes and at room temperature and standard atmosphere. The influence of the relative concentration of each Ternary Solution component on the final pattern topology has been studied, allowing to create a ternary diagram that can be used as a guide for rationally designing PMMA patterns over large areas, and for better understanding the behaviour of the formulated TSs.

The size of, and the relative distance between, the formed patterns appears to be an exponential decay function of the relative concentration of the Auxiliary Solvent in the Ternary Solution, suggesting that the self-assembly processes of PMMA occurring within the Sublimating Substance matrix can be described by well-known polymer chains diffusion laws. This hypothesis is currently being verified.

On the basis of the above mentioned findings, a qualitative explanation of the phenomena underlying the formation of the patterns has been given. In particular, it is proposed that the Sublimating Substance crystallites exert an effective templating effect, and that the transition from continuous fibres to dots is governed by the SS/TM layer thickness left on the substrate after the Auxiliary Solvent has evaporated.

Finally, some of the developed patterns have been used as masks for ion-etching-based lithographic processes, evidencing a good transfer of the mask features to the underlying substrate (Si/native SiO_x_) with noticeable aspect ratios. These results highlight the potential of ASB-SANS-generated patterns in applications like photovoltaics, sensing and plasmonics.

## Methods

### Materials and instruments

Poly(methylmethacrylate) (PMMA) (number-averaged molecular weight, Mn = 15.000 Da), para-dichlorobenzene (PDCB) (99 + %), CHCl_3_ (99.9 + %) were purchased from Aldrich and used as received. p-Doped Si/SiO_x_ substrates (100) were purchased from ITME (Poland). To ensure a constant temperature of the substrates onto which the nanostructures were developed, a copper plate in thermal contact with the secondary water tank of a Haake thermostated bath (the secondary water tank was mechanically decoupled from the main one in order to ensure a vibration-free thermostated environment) was used as a support for the substrates. SEM photos were taken with a Carl Zeiss Supra 40 Scanning Electron Microscope. The experiments were carried out at room temperature and pressure, under a fume hood when needed.

### Preparation of substrates

Doped Si/SiO_x_ (native oxide layer) substrates were used to facilitate the subsequent scanning electron microscopy (SEM) investigation. At the first usage of substrates, they were cleaned with soap, acetone and isopropyl alcohol, respectively. When re-used, the substrates were cleaned by sonicating for 5 min in CHCl_3_, then with isopropyl alcohol, dried and dipped in fresh CHCl_3_. In cases when this procedure did not ensure a thoroughly clean substrate, the substrates have been immersed in a solution composed by H_2_O_2_:H_2_SO_4_, 1:1 V/V for 30 minutes, after which they have been washed with plenty of distilled water and left for 30 min in an oven (standard atmosphere) at 300 °C. These procedures allowed to obtain a CHCl_3_ spreading behaviour similar to that of the pristine Si/SiO_x_ substrates. All the substrates have been dried with nitrogen gas prior to the TS drop-casting step.

### Preparation of ternary solutions and drop casting

A stock solution was prepared with 1 mg of PMMA dissolved in 1 mL of CHCl_3_ in a closed vial. After sonication for 5 min the stock solution was left stirring for one day, and then filtered (PTFE filter, 0.45 μm) to eliminate possible particulate. From the stock solution, sixteen different ternary solutions were prepared, each having different relative amounts of the three components, as described in Table 1, to explore the effect of the composition changes on the developed TM patterns. After these operations, all the prepared solutions appeared limpid and homogeneous.

The Ternary Solution have been carefully drop-cast onto the substrate by means of a Pasteur pipette, taking care for constantly dropping (10 ± 1) mg of solution (as determined from 75 sampled TS droppings onto the substrate put on an analytical balance, carried out over representative TS compositions among those reported in Table 1) onto an approximately 1 cm^2^ surface. The drop casting was carried out at room temperature, about 24 °C, and ambient pressure, depositing the Ternary Solution onto the substrates in thermal contact with a vibration-free thermostated water bath kept at (23.0 ± 0.2) °C (as measured via a Type J thermocouple). After drop-casting the Auxiliary Solvent was allowed to completely evaporate. At the end of the AS evaporation process, the resulting solid, crystalline film (Sublimating Substance incorporating the Target Material, see step v of Fig. 1) was put inside a small Petri dish (diameter 3 cm, height 7 mm), not sealed, to set up a closed chamber into which sublimation conditions as close as possible to equilibrium (i.e., to saturate the atmosphere surrounding the substrate with SS vapours) are established. Substrates were then left to rest at least 12 hours to ensure the complete sublimation of the SS. After this period the resulting patterns have been imaged by SEM. In some cases the samples have been re-imaged by SEM also after several months.

### Determination of the topological parameters

As the manual procedure followed for depositing the TS onto the substrate brings some unavoidable variability, for each TS composition (Table 1) the nanopatterns growth experiments have been repeated from 20 to 50 times before results were compared and examined. Once the substrates were ready, pictures of each sample have been taken via optical microscope and SEM. The free Gwyddion software^43^ has been used on SEM images to analyze pattern topologies in terms of Pattern Width and Inter-Pattern Distance. As the analyzed topological parameters were all accessible by SEM imaging, no determination of the nanopatterns vertical thickness (for example using AFM or White Light Interferometry) has been carried out.

### Silicon etching process

The pattern transfer of nanostructures in the bulk silicon substrate has been achieved by Dry etching protocol in Inductively Coupled Plasma-Reactive Ion Etching (ICP-RIE). The patterned PMMA of type M (see Table 1) has been exploited directly as the mask (nanodots thickness: about 70–75 nm, see Fig. S2 in the Supporting Information); the suitable process parameters have been set in order to maximize the aspect ratio of the obtained structures (gases: SF_6_, C_4_F_8_, Ar, 60:30:10 sccm. P: 8 mTorr. Power: 400 W. The etch process time was 100 s). The etching step has been followed by O_2_ plasma so as to remove the mask remainder.

## Electronic supplementary material

Supplementary Information
