# Supplementary material for: Controlled self-organization of polymer nanopatterns over large areas
Source: Sci Rep. 2017 Sep 5;7:10526. doi: 10.1038/s41598-017-09463-z (PMC5585330; doi:10.1038/s41598-017-09463-z)
Supplement: Supplementary file 1 — Supplementary Information [file 41598_2017_9463_MOESM1_ESM.doc]

**Controlled self-organization of polymer nanopatterns over large areas**

Ilknur Hatice Eryilmaz^1^, John Mohanraj^1§^, Simone Dal Zilio^2^, Alessandro Fraleoni-Morgera^1^*

1: FlexTronix Laboratory, Dept. of Engineering and Architecture, University of Trieste - V. Valerio 10 - 34100 Trieste (Italy)

2: IOM-TASC CNR, Strada Statale 14 km 163,5 - 34149 Basovizza, Trieste (TS), Italy

§: JM is now at the Advanced Functional Polymers Laboratory, Department of Macromolecular Chemistry I, University of Bayreuth - 95440 Bayreuth (Germany)

*: email: afraleoni@units.it

**Supporting Information**

**Figure S1** - a), b): SEM images of PMMA nanopatterns of type E, I, respectively, at low magnification.


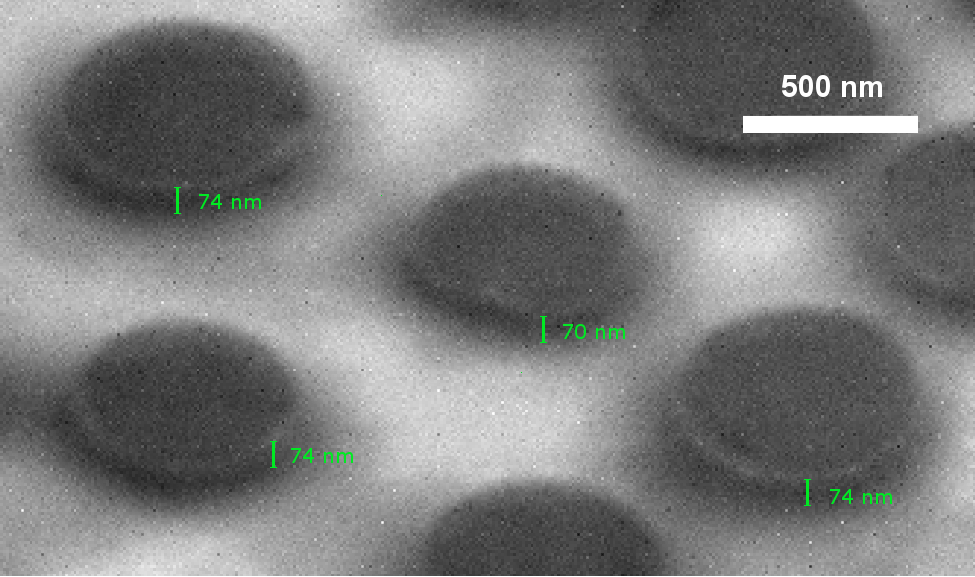


**Figure S2** - SEM image of the type M patterns used as lithographic mask for the ICP-RIE treatment, evidencing the thickness of the patterns.
